# Tooth Tablets

**Published:** 1875-06

**Authors:** 


Tooth Tablets.—Still inquiries come to us for Dr. Lyon's address. Scores of persons assure us that they never heard of this valuable dentifrice until we called their attention to it. To such as fail to find it at the druggist's, we would say, tell your local apothecary to send to New York and obtain a lot of it. It will all be sold, we can promise, as we do not mean to give up the work of urging its merits, yet awhile and we think our influence is sufficient to induce thousands of sensible folks to use it daily for the remainder of life. But such as prefer to write to Dr. Lyon, can reach him by letter. His full address is—Dr. I. W. Lyon, No. 36 Vesey Street, New York City. He will mail a box of his Tablets, postage paid, for 50 cents, or will send a small trial-box for 10 cents.

---

The imagination is of so delicate a texture that even words wound it.

